# Climate Change, Health and Informatics: a Narrative Review

**DOI:** 10.1055/s-0046-1816685

**Published:** 2026-11-03

**Authors:** Rosario Maria Torres Santos-Olmo, Angel Abad Revilla, Arianna Dagliati, Daniele Pala, Riccardo Bellazzi, Fernando Martin-Sanchez

**Affiliations:** 1 Department of Medical Informatics. Hospital Universitario “La Paz”. SERMAS. Madrid. Spain. LIMA (Laboratory of Advanced Medical Informatics). Instituto de Investigación Sanitaria “IdiPAZ” MadridSpain; 2 Sustainable Development Office. Hospital Universitario “La Paz”. SERMAS. MadridSpain; 3 Department of Electrical, Computer and Biomedical Engineering, University of PaviaItaly; 4 Department of Management, Information and Production Engineering, University of Bergamo, BergamoItaly

**Keywords:** Health, Public health, Environment, Artificial intelligence, Climate Change

## Abstract

**Objectives** : Human health is influenced by genetic, socioeconomic, environmental, and behavioral factors. Climate change represents one of the greatest threats to global public health in the 21st century, impacting both communicable and non-communicable diseases, and challenging the resilience of healthcare systems. Information and Communication Technologies (ICTs) and Artificial Intelligence (AI) applied to health play a dual role: while they contribute to climate change, they also offer essential tools to address it through public health and healthcare management strategies. To explore the intersections between climate change, human health, and informatics by analyzing how digital technologies—particularly AI and ICTs—can contribute to mitigating the health impacts of climate change, promoting sustainable behaviors, and strengthening the adaptive capacity of healthcare systems, while also addressing the sustainability challenges these technologies entail.

**Methods** : A narrative review was conducted to explore how digital transformation interacts with climate change and its subsequent effects on human health. A comprehensive search of the Scopus and PubMed databases yielded 1,130 articles from 2015 to 2025. After screening for relevance, 24 key studies were selected, providing insights into distinct phases of research evolution in this field. Seven papers were then added manually as they were considered relevant.

**Results** : Digital transformation (ICTs, AI) in health has driven sustainable innovations, exemplified by projects such as PULSE and BRAINTEASER, which leverage technology to prevent health risks, optimize interventions, and manage urban resources. The development of smart and eco-cities highlights the transformative potential of digital technologies to create sustainable urban environments, provided they are supported by policies promoting renewable energy, circular economies, and emissions reductions. However, digital transformation entails significant sustainability challenges, including energy consumption and emissions, water usage, electronic waste and resource depletion. The need to strike a balance between the potential of ICTs and AI to improve public health and their environmental impact is strongly emphasized throughout the article. Although well-established strategies exist to mitigate these effects—such as energy efficiency, circular economy models, the use of clean energy, remote work, and telemedicine—there is still a lack of standardized protocols and global policies to enable their effective implementation.

**Conclusion** : ICTs, and particularly AI, play an ambivalent role in the face of health and climate change: they can be part of the problem or part of the solution. To maximize their positive impact and reduce their ecological footprint, it is urgent to establish responsible policies, sustainable models of technological development, and standardized procedures to guide their application in the fields of health and the environment.

## 1. Introduction

Human health is primarily determined by genetic and biological factors, socioeconomic conditions, environmental influences, individual behaviors, and access to healthcare services. Protecting both individual and public health requires the preservation of a healthy environment.

Climate change has emerged as one of the most significant threats to global public health in the 21st century. Its impact on the social, environmental, and structural determinants of health is becoming increasingly evident. Climate change contributes to the rise in infectious, respiratory, cardiovascular, and mental illnesses [ 1 ], among others, and challenges the resilience of healthcare systems. These effects occur through the alteration of weather patterns, the intensification of extreme meteorological events, and environmental degradation.

Environmental factors account for approximately 23% of global mortality [ 2 ]. The World Health Organization (WHO) estimates that climate change will result in an additional 250,000 deaths per year between 2030 and 2050 due to malnutrition, malaria, diarrhea, and heat stress [ 2 ]. The health impacts of climate change are both direct—such as heatwaves—and indirect, through poor air and water quality, an increase in vector-borne diseases, and disruptions to socioeconomic systems caused by famines and natural disasters.

Climate change is driven by the increasing concentration of greenhouse gases (GHGs), primarily resulting from human activities such as fossil fuel combustion, deforestation, and intensive agriculture. While the natural greenhouse effect is essential for maintaining temperatures conducive to life on Earth, excessive emissions of carbon dioxide (CO _2_ ), methane (CH _4_ ), and nitrogen oxides (N _2_ O) are intensifying global warming and altering the climate. The primary sources of GHG emissions include electricity generation (28%), transportation (25%), industry (21%), agriculture and livestock farming (14%), information and communication technologies (ICTs) (6%), and buildings (6%) [ 3 ].

ICT interacts with health and climate change in a dual manner. On one hand, it contributes to climate change through GHG emissions, high energy consumption, waste generation, and significant water usage. On the other hand, the expansion of ICT has transformed the healthcare ecosystem through the development of health informatics methods and digital health solutions. This field encompasses the use of digital tools such as mobile applications, wearable sensors, artificial intelligence (AI), clinical information systems, telemedicine platforms, and early warning systems to enhance well-being, prevent diseases, support diagnosis and treatment, facilitate research, and improve healthcare management. In the current climate context, this discipline is emerging as a key tool for monitoring environmental exposures, anticipating and modeling risks, personalizing interventions, and enabling coordinated responses to emergencies [ 4 ].

This narrative review aims to explore the intersections between climate change, human health, and informatics, focusing on how the latter can contribute to mitigating the health impacts of climate change, promoting sustainable behaviors, and strengthening the adaptive capacity of healthcare systems. Digital technology can reduce its environmental impact through more efficient designs that optimize processes, minimize resource consumption, and reduce waste generation.

This study is based on a literature review of recent scientific publications, particularly articles published between 2015 and 2025, which examine the connections between climate change, human health, and informatics.

The choice of a narrative review approach is justified by the need to integrate diverse perspectives (public health, technology, environmental science, and ethics) within a comprehensive and accessible framework. Unlike systematic reviews, which focus on specific research questions, this review provides a broader reflection on the role of health informatics in the context of a global ecological crisis.

The following sections, building upon the conceptual framework introduced here, will address, as depicted in Figure 1 :

**Figure 1.**
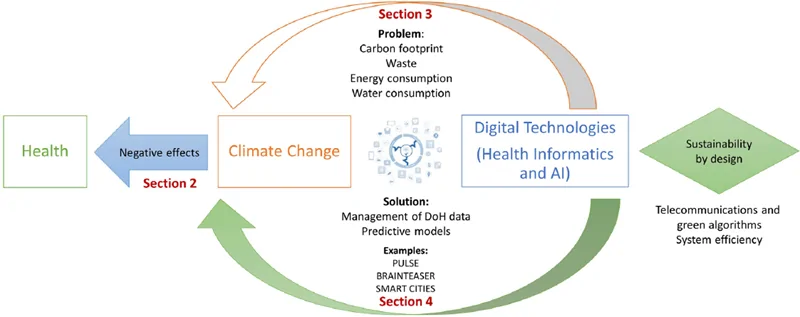
Intersections between climate change, human health, and informatics. DoH stands for Determinants of Health.

- The effects of climate change on human health (Section 2);
- The main impacts of digital technologies, including health informatics and AI on climate change and, consequently, on human health (Section 3);
- The potential for health informatics and AI as tools for climate change mitigation and adaptation, along with the ethical, regulatory, and sustainability challenges associated with this approach (Section 4).

The article concludes with a discussion on future research directions and policy recommendations aimed at fostering a more resilient, equitable, and environmentally responsible healthcare model.

## 2. Impacts of Climate Change on Human Health

Climate change affects human health through multiple, complex, and context-dependent mechanisms that vary by region, demographic profile, and population vulnerability [ 5 6 7 ]. To address this, various strategies and tactics aim to reduce the healthcare sector's contribution to climate change. By implementing these strategies, healthcare systems can significantly lower their carbon footprint and move toward a greener model powered by renewable energy. The healthcare sector can lead the transition toward net-zero emissions by 2050. In doing so, it can help reduce the patient burden that global warming would otherwise increase.

This section explores the key pathways through which climate change affects health, with a focus on communicable and non-communicable diseases, mental health, food security, and social inequalities. It also highlights findings from the Global Burden of Disease (GBD), a major effort to quantify health loss across different regions and time periods in order to improve healthcare systems and reduce disparities.

### 2.1 Communicable Diseases and Climate Change

Climate change alters the habitats of vectors such as mosquitoes, ticks, and rodents, reshaping the geographic and seasonal patterns of diseases like dengue, malaria, West Nile virus, and Lyme disease [ 8 ]. Higher temperatures, irregular rainfall, and flooding promote vector proliferation and enhance their transmission capacity [ 9 ]. At the same time, extreme events such as hurricanes and droughts disrupt access to clean water and sanitation, increasing the risk of infectious disease outbreaks [ 10 ].

### 2.2 Non-Communicable Diseases and Mental Health

Climate change also contributes to rising rates of non-communicable diseases, including cardiovascular, respiratory, and metabolic conditions [ 5 ]. Heatwaves raise the incidence of myocardial infarctions [ 6 ] and strokes, while air pollution—exacerbated by urbanization and weather patterns—worsens asthma and chronic obstructive pulmonary disease (COPD).

Mental health has also suffered significantly [ 7 ]. Post-traumatic stress following disasters, climate-related anxiety (eco-anxiety), depression linked to the loss of livelihoods or habitats, and rising suicide rates in drought-stricken areas have been documented across the globe. According to the GBD Study 2021, approximately 746,000 deaths by suicide occurred that year, serving as an important indicator for tracking mortality trends [ 11 ].

### 2.3 Food Security, Nutrition, and Health

Climate variability disrupts agricultural production, affects food availability and nutritional quality, and undermines food security. Droughts and floods destroy crops, reduce livestock productivity, and damage fisheries, creating widespread food insecurity. These impacts can lead to malnutrition, micronutrient deficiencies, and related diseases, particularly among children, pregnant women, and the elderly.

A review presented at the EGEA 2023 conference [ 12 ] emphasized that plant-based diets rich in fruits and vegetables not only reduce the risk of cardiovascular diseases and type 2 diabetes but also have a lower environmental impact compared to other dietary patterns—except for the high water demand of some crops. Promoting these sustainable diets could play a key role in achieving the Sustainable Development Goals (SDGs).

### 2.4 Differential Vulnerability and Social Determinants

Climate change does not affect all populations equally. Vulnerable groups—including children, the elderly, women, persons with disabilities, Indigenous communities, migrants, and people living in poverty—face higher exposure risks, possess lower adaptive capacity, and encounter more barriers to accessing protective resources [ 13 ].

Structural health determinants such as education level, gender, geographic location, and socioeconomic status influence exposure to extreme events, environmental quality, and access to digital technologies for adaptation and mitigation [ 14 ]. Policymakers must integrate equity considerations into all health and climate strategies.

## 3. Environmental Challenges of Healthcare, ICT and Health Informatics in Relation to Climate Change

Researchers have extensively documented the direct and indirect impacts of climate change on human health over the past two decades [ 15 ]. Understanding the relationship between climate change, human health, and informatics demands a multidisciplinary approach that combines concepts and methods from public health, environmental science, health informatics, and ethics.

In today's society, ICTs and informatics play a multifaceted role, offering significant benefits in terms of efficiency, accessibility, and innovation. However, it also presents major sustainability challenges due to its high consumption of energy [ 16 ], water, and natural resources, as well as its carbon emissions [ 17 ] and electronic waste generation.

Healthcare systems hold a unique position in this landscape. While they serve on the front lines in addressing climate-induced medical needs, they also emit considerable amounts of carbon, consume large quantities of energy and water, and generate electronic waste [ 18 ]. These systems must therefore confront a key dilemma: how to deliver high-quality care while responding to increasing climate-driven demand and simultaneously reducing their own substantial contributions to the climate crisis. Hospitals in particular consume vast amounts of energy and produce significant waste [ 19 ]. Although healthcare must care for patients and uphold the principle of “do no harm”, stakeholders have only recently begun recognizing the sector as a contributor to climate change [ 20 ]. Current estimates suggest that healthcare accounts for approximately 4.4% of global carbon emissions, with national ranges between 4% and 8.5% [ 21 ].

This section examines the intersection between ICTs and climate change in healthcare, focusing on the environmental burden of these systems [ 22 ].

### 3.1 Energy Demand and Carbon Footprint of ICT

In 2017, ICTs accounted for roughly 7% of global energy consumption, and forecasts suggest a 30% increase by 2026, reaching 3% of total electricity use in the European Union [ 23 ]. AI systems, particularly deep learning models, require substantial computational resources and high energy use during both training and inference phases [ 24 ]. A 2019 study by Strubell *et al.* estimated that training a single large AI model could emit as much carbon dioxide as five cars over their entire lifetime [ 25 ].

ICT-related carbon emissions currently account for 3% to 4% of global CO _2_ output—already surpassing the aviation sector—and may rise to 14% by 2040 if left unaddressed [ 26 ]. Notably, generative AI searches can consume up to 30 times more energy than a standard web search [ 27 ].

Beyond operational emissions, ICT systems also affect the global carbon cycle through device production, transport, and disposal. Semiconductor and microchip manufacturing processes consume high amounts of energy and rely on raw materials extracted from environmentally sensitive regions, often under weak environmental regulations, further intensifying their ecological footprint [ 28 ].

### 3.2 Water Consumption in ICT Operations

ICT systems consume large volumes of water, especially in data centers that rely on intensive cooling systems. Companies such as Google, AWS, and Meta reported daily water usage as high as 51.78 million liters in 2020. A single query to a language model such as ChatGPT may consume between 0.01 and 0.1 liters of water [ 29 , 30 ]. In water-stressed regions, such usage increases local scarcity and may fuel resource conflicts [ 31 ]. These concerns underscore the need to design ICT infrastructure with water sustainability in mind.

### 3.3 Electronic Waste and Resource Depletion

The rapid growth of the ICT sector has led to a sharp increase in electronic waste (e-waste), which may reach 43 million tons annually by 2030 [ 32 ]. This trend reflects the short lifespan of digital devices, low recycling rates, and fast-paced technological obsolescence. E-waste contains toxic substances such as lead, mercury, and cadmium, which pose serious risks to both human health and biodiversity when released into the environment [ 32 33 ].

In addition, e-waste represents a loss of rare and critical metals whose extraction involves severe environmental degradation and often generates social conflict. Although recycling can recover some of these valuable materials, global implementation remains limited [ 34 35 ]. Social repercussions also deserve attention: informal recycling—especially common in low-income countries—exposes vulnerable communities to hazardous pollutants and exacerbates structural inequalities [ 36 ]. A transition toward a circular economy is essential to mitigate these impacts.

### 3.4 Climate Impacts of AI in Healthcare

The Intergovernmental Panel on Climate Change (IPCC) has stressed the urgent need to mitigate climate change and adapt to its unavoidable consequences [ 37 ]. As healthcare contributes a significant share of global greenhouse gas emissions, it must play a central role in climate action [ 21 ].

The rapid growth and widespread adoption of AI in healthcare have raised concerns regarding its environmental footprint [22. Deep learning models, in particular, demand extensive computational power and energy during both training and inference [24. According to Strubell et al, training a large AI model can emit as much CO _2_ as five vehicles over their lifespan [ 25 ]. The healthcare sector, responsible for 4.4% of global emissions, must confront the added burden of AI-related energy consumption. Large datasets, complex algorithms, and frequent model updates intensify the energy demands of AI systems.

Data centers—essential to AI infrastructure—further contribute to the sector's carbon footprint [ 38 ]. Their servers, cooling systems, and networking equipment require continuous power, often derived from high-emission sources [ 39 ]. As AI systems grow more complex and data-hungry, data centers will likely consume even more energy, worsening their environmental impact. The International Energy Agency (IEA) reported in 2020 that data centers accounted for about 1% of global electricity consumption, a figure expected to rise with increasing AI and digital service demands [ 40 ].

Another environmental concern involves the rise in electronic waste and depletion of natural resources caused by healthcare AI [ 34 ]. Discarded electrical and electronic devices often contain hazardous materials—like lead, cadmium, and mercury—that can harm human and environmental health if improperly disposed of. Rapid tech development and demand for computing hardware lead to frequent upgrades and accelerated obsolescence [ 41 ]. Mishandling e-waste can release toxic substances, posing serious environmental and public health threats.

AI development and deployment also cause indirect environmental effects. Mining rare earth elements and other materials essential for AI hardware may destroy habitats, reduce biodiversity, and pollute water supplies [ 42 43 ]. The transportation and logistics required in global supply chains for AI components and devices generate greenhouse gas emissions [ 26 ]. As healthcare continues to integrate AI into its core operations, stakeholders must evaluate and address these environmental consequences through effective mitigation strategies.

## 4. ICT in support of environmental sustainability

While ICT operations may have a damaging environmental impact, the same technologies, if properly used, can be beneficial to humans and even improve sustainability reducing pollution and damaging exposures. Finding the right trade-off between positive and negative environmental effects of ICT operations especially AI, can be a complicated task. This section aims to highlight the potential of AI and ICTs to improve environmental sustainability, and at analyzing the complex interplay between the search for sustainability and the environmental impact of technological advancements.

### 4.1 Literature Search

Although both AI and sustainability are not new concepts, attention to the link between the two topics significantly increased in the last years, as a consequence of the advent of Large Language Models and the consequent widespread use of AI systems. After a first search in the literature, we determined that the heterogeneity and the rapid change in research directions on the interplay among AI, environment, and health, were too vast to allow for a systematic review, therefore a narrative review was undertaken.

Articles were gathered both through manual selection and database search. In particular, the Scopus and PubMed databases were searched through two different queries:

Query1: (ICT OR “artificial intelligence”) AND “sustainability” AND “public health”

Query 2: (ICT OR “artificial intelligence”) AND “sustainability” AND environment* AND health

The search extracted 1,130 articles from both databases, after removing the duplicates and filtering for those published in the last ten years (2015-2025), a choice was made considering the relatively novel interest in the topic. Seven papers were then added manually as they were considered relevant even though they were not selected by the queries. All articles were then screened to determine their relevance to the scope of this review, leading to a final list of 24 papers. In particular, only the papers treating the sustainability and/or environmental impact of AI or ICT technologies as the main focus were kept after a rapid assessment of their topics, in line with the scope of our narrative review.

Several phases of the evolution of research in this context were then identified through an inspection of the selected publications, which in part presented the use of AI to model the effects of environmental exposures or to optimize industrial or clinical processes, and in part focused on the construction of smart cities or the direct impact of AI on climate change.

### 4.2 Environment and Population Health

Numerous studies present the development of descriptive and predictive models focused on the effects of environmental exposures and climate on human health. With the fast technological development of the last few years leading to a changing lifestyle for most of the world's population, increasing attention has been given to air pollution and lifestyle on numerous physical conditions and on mental health. These studies usually focus on disease onset prediction, progression modelling and/or population stratification. The application of AI and ICTs has been demonstrated to be beneficial to public health as it helps to better characterize the complex link between environmental exposures and health, paving the way to optimized prevention and intervention strategies. Patient stratification or clustering has been proposed as a recommended way to optimize health interventions improving public health outcomes and, if performed properly, sustainability. For example, Wong *et al.* [ 44 ] conducted a review about the use of AI in ophthalmology to reduce environmental impacts related to patients' visits and treatments, in which they highlighted, among other things, the usefulness of applying AI for patients' stratification to reduce pollution and waste generation. Other reviews [ 45 46 ] discuss the potential of applying AI to improve waste reduction and energetic efficiency, collecting studies that show that, globally, AI has good performances predicting biomass generation, energy outputs, solid waste generation and recycling, although some challenges are still present. From the quantitative point of view, the performances vary depending on the specific study and environmental setting but are generally high. For example, Pitakaso *et al.* [ 47 ] propose an AI-based system to classify waste types and distinguish infective ones that could be used as an automated system to optimize waste management processes, reaching classification accuracies of almost 98%.

In the last decade, several academic projects focusing on these topics have been funded. Two examples of these projects are the PULSE and BRAINTEASER projects, both funded by the European Commission under the framework HORIZON 2020. Both these projects have produced studies and tools centered on the use of AI in improving the prevention of health outcomes associated with external exposures, to create optimized and sustainable public health frameworks to improve population health.

More in detail, PULSE (Participatory Urban Living for Sustainable Environments) implemented a community-based framework aiming at using data science and AI to assist patients with asthma, type 2 diabetes and cardiovascular diseases in big cities. Urban environments are peculiar test beds for this kind of research and optimizing public health strategies and interventions can lead to a significant reduction of environmental impact. PULSE aimed to contribute to sustainability mainly through the following activities:

Construction of models of pollution and climate trends that can be used to estimate the levels of environmental risk and plan optimized interventions [ 48 ];

Spatial and population clustering can be used to optimize health management by studying large urban areas subdivided into similar macro areas [ 49 50 ];

Simulation tools that can provide projections on the effects of interventions, reducing the possible waste of resources [ 51 ];

Construction of an approach that involves the entire community, with a centralized data management system that requires fewer resources than using separate ones, and education strategies for both the population and the policy makers [ 52 ].

BRAINTEASER (BRinging Artificial INTelligencE home for a better cAre of amyotrophic lateral sclerosis and multiple SclERosis) is another project that integrates large clinical datasets with novel patient-generated and environmental data collected using low-cost sensors, apps, and innovative ICT solutions. The collected data have enabled the development of AI tools capable of early risk prediction of disease onset, rapid progression, and adverse events [ 53 54 ].

BRAINTEASER's technical solutions, developed within the project, follow agile and user-centered approaches that address the technical, medical, psychological, and societal needs of specific users. More specifically, BRAINTEASER collects environmental data from multiple sources, including public datasets, fixed air quality sensors, and wearable sensors used by patients. These data have demonstrated the feasibility of computing personal exposure with fine granularity, assessing parameters such as average time spent indoors/outdoors and weekday/weekend activity patterns. Specific models illustrate how personal exposure can be calculated by combining patients' GPS positions with interpolated air pollution measurements derived from air quality sensors. For example, some models rely on a graph-based representation of urban spaces (where nodes represent road intersections and links represent streets connecting those intersections) and Land Use Regression (LUR) methods to compute precise pollutant concentrations, such as PM2.5.

BRAINTEASER focuses on delivering cost-effective and accessible solutions by leveraging cloud-based software and affordable mobile and portable sensor technologies for personalized medicine. The project emphasizes computationally efficient approaches that can be implemented in various contexts, ensuring broad applicability. Following an open-science approach and making available a rich and shared data ecosystem (including through initiatives like the CLEF challenges 1 ), BRAINTEASER makes AI models available to the scientific community as research tools. The most effective models are also selected for further open cloud access or potential commercial development. Thanks to a rich and shared framework, BRAINTEASER provides evidence of the effectiveness of using AI in healthcare pathways - not only during the project development, but also for future research. Throughout the project course, BRAINTEASER offered several examples for the use of AI in real clinical settings for amyotrophic lateral sclerosis and multiple sclerosis progression [ 55 56 ]. Additionally, its outcomes will offer a coherent and integrated set of recommendations for public health authorities, paving the way for extending this approach to other neurodegenerative diseases.

Projects like PULSE and BRAINTEASER are examples of how AI can improve public health through risk assessment and population stratification. However, the development and application of AI models must be done keeping in mind that ICT technologies themselves have an impact, which can be reduced only through responsible use of energy and the creation of sustainable strategies. A recent review by Ueda *et al.* [22 focusing on the role of AI in healthcare on climate change has highlighted the importance of using sustainable energy practices and focusing on policymaking and governance to minimize the damage caused by ICT technology use. An older study by Hadley *et al.* [ 57 ], focusing on the adoption of AI in low-income areas and its sustainability, also emphasizes the same concepts, adding the benefits of selecting low-maintenance AI models and forming local onsite personnel to reduce travel and consumption of resources.

### 4.3 Smart Cities and Smarter Eco-Cities

The application of AI models and technology in public health has led to the development of new concepts and new strategies to improve health and quality of life. For instance, through frameworks such as PULSE, which joins together AI, modern sensor technology, and education of public health operators and policymakers, the concept of Smart Cities was generated. Smart cities are cities where traditional networks and services are made more efficient with the use of digital solutions for the benefit of its inhabitants and business [ 58 ]. This is obtained through the use of technology, Internet of Things (IoT) and AI models to improve public services and urban planning in order to improve quality of life, reduce pollution, and mitigate health risks. With the majority of the world's population living in cities, smart cities represent a very important model of sustainable development, where, in an ideal scenario, the advantages of using ICT technology outnumber the disadvantages from the point of view of the environmental impact. Recent research has shown the high potential of the application of AI for the improvement of waste management processes, water quality monitoring and hazardous exposures monitoring [ 47 59 ]. Urban planning can also be efficiently optimized through the application of AI, Artificial Intelligence of Things (AIoT) and predictive modelling, which can help in several processes, such as public health monitoring and low-carbon materials selection [ 60 ].

However, the development of smart cities does not come without significant challenges. A recent review by Bibri *et al.* [ 61 ] presents the intricate world of “smarter eco-cities”, i.e. a new paradigm characterized by the integration of innovative technologies and environmental solutions. In this review, the authors deeply inspect the complex links between data-driven technologies, environmental strategies and urbanism paradigms that are at the basis of the idea of smart eco-cities. However, they also highlight some gaps in understanding how these concepts are intertwined. This study emphasizes the importance of implementing AI in a responsible and sustainable way, using renewable energy as much as possible, optimizing energy consumption, improving environmental monitoring and encouraging circular economy.

### 4.4 Direct Relation between AI and Climate Change

Last year's fast-increasing interest towards sustainability in the use of ICTs and AI has been largely driven by a rising awareness about climate change. Global warming is making extreme weather events more common, such as floods and dangerous heat waves that can expose the most sensitive parts of the population to a significant risk of cardiovascular events [ 62 ]. AI and in general ICTs can help mitigate these risks through practices such as the prediction of extreme events, the identification of susceptible areas and populations, and the creation of decision support systems that the policymakers can use to improve urban planning and intervention strategies [ 63 64 65 ]. However, ICTs themselves can contribute to global warming through pollution generated by maintenance processes and e-waste. A recent study by Wang *et al.* 66showed that generative AI models alone are estimated to be responsible for high quantities of e-waste, potentially reaching a total accumulation of 1.2–5.0 million tons during 2020–2030 [6671]. In the same article, however, circular economy strategies are demonstrated to be efficient in reducing e-waste significantly. Together with the circular economy, most studies propose several solutions to the problem of carbon footprint due to ICTs, especially in healthcare: the use of simpler models, the choice of renewable energy (where AI can also be used to optimize the waste reduction process [ 67 ]), the use of telemedicine to reduce in-person visits and improve screening and prevention, the recycling of water for cooling systems, and the application of AI for environmental monitoring [ 22 , 68 – 70 ]. These strategies cannot be properly executed without dedicated policies and standardized procedures, which, as pointed out by Lokmic-Tomkins *et al.* [ 71 ], are currently lacking. Validated methods to assist stakeholders in decision-making considering carbon footprint are necessary to reach the sustainability goal.

Despite the lack of standardized policies and procedures, the global research panorama shows encouraging signs. For example, the Journal of the American Medical Informatics Association (JAMIA) published a special issue on climate change and informatics in 2022, which included numerous papers focused on assessing the impact of informatics on climate change and proposing solutions to reduce the risk of harm related to it [ 72 ]. This special issue, on the one hand, depicts a global panorama of the field, with articles such as the one by Rahimi-Ardabili *et al.* [ 73 ] which show that digital health can help manage climate change, but theoretical results usually do not turn into real-world evaluation. On the other hand, some authors actively propose intervention strategies, such as Sarabu *et al.* [ 74 ] who performed a pilot survey of informatics professionals' perspectives on climate change and health, or Sittig *et al.* [ 75 ] who propose a framework to illustrate how clinical informatics can help reduce healthcare's environmental pollution.

Also, lots of recent research projects are oriented towards reaching sustainability goals and/or responsibly applying technology taking into account its potential carbon footprint. The European Commission, for example, started a program called LIFE [ 76 ], that is entirely dedicated to the shift towards clean energy and the implementation of sustainable tools and techniques to improve public health, quality of life and the economy, 2 while also improving the quality of the environment. Another important initiative in this field is the European Green Digital Coalition, an initiative of companies, supported by the European Commission and the European Parliament, based on the request of the EU Council, which aims to harness the enabling emission-reducing potential of digital solutions to all economic activity sectors. Some of the largest ICT-based companies, such as Google, AWS and Microsoft, are also developing strategies to reduce their water consumption and pollution production.

## 5. Discussion

Climate change poses a significant threat to human health, as it can directly and indirectly lead to increased risk factors or exacerbation of numerous diseases. People's health can be safeguarded through mainly two main strategies: Mitigation (i.e., the reduction of pollutants emissions and carbon footprint), and Adaptation (i.e., managing existing risks and fostering resilience to the impacts of climate change). In the context of digital transformation of health, ICTs, including AI, play a dual role. While they contribute to GHG emissions due to energy consumption, data center cooling, and waste generation, they also offer innovative solutions to reduce their environmental impact and improve health. For this reason, finding the right trade-off between these two aspects of the development and implementation of ICTs has quickly become one of the most important challenges of the present times.

As shown with our literature search and through the recent examples of academic projects such as PULSE and BRAINTEASER, consistent research is being carried out on these topics, showing the high potential of the use of ICTs, and in particular AI, to improve public health, support policy makers' decision making, optimize industrial processes and improve waste management. These new concepts gave birth to new paradigms, such as the so-called smart cities: ideal urban environments in which technology is integrated in most public services and urban planning processes, leading to the creation of an efficient and clean environment. Several of the selected studies show successful examples of the application of AI to improve processes such as waste management, water conservation, and public health intervention design. Plus, numerous studies demonstrate the effectiveness of some strategies aimed at reducing the impact of AI models, such as:

Energy efficiency improvement: optimizing energy using efficient cooling systems, recycling when possible, and reducing complexity and the number of physical servers;

Use of renewable energy: powering data centers and ICT infrastructures with renewable energy sources can significantly reduce carbon emissions;

Hardware optimization: utilizing energy-efficient hardware and extending the lifespan of devices through a circular economy help lower the carbon footprint;

Promotion of remote work and video conferencing: encouraging telework and video conferencing reduces the need for travel, thereby decreasing carbon emissions associated with transportation for professionals, suppliers, clients, etc;

Promotion of telemedicine: as for telework, telemedicine has also been demonstrated to have a beneficial impact on the environment, as well as on patient's health, which can be monitored more efficiently even without in-person visits;

Smart resource management: implementing practices for efficient resource management, such as automatic shutdown of unused equipment and optimization of data storage, contributes to reduced energy consumption;

Application of AI to increase climate change adaptation: AI can be a powerful tool to identify individuals most vulnerable to climate change and design actions to minimize the risks associated with global warming and extreme weather events.

In spite of these commonly recognized aspects, there is still a general lack of standard protocols and global policies to encourage the large-scale application of these mitigation processes. Such elements are necessary to organically create ICT systems that can maximize efficiency while minimizing environmental impacts coming both from their own production and usage and most other human activities.

## 6. Conclusion

The relationship between ICTs and sustainability presents a complex dilemma involving benefits and challenges. This review shows that while ICTs, including AI, offer significant opportunities to enhance efficiency, accessibility, and sustainability in areas such as public health and environmental management, it also raises substantial concerns due to their intensive consumption of energy, water, natural resources, and the generation of electronic waste.

ICTs, and especially AI, can be a powerful tool to improve health and quality of life, and even reduce pollution and contribute to the mitigation of the climate change problem. However, their development and applications must follow specific responsible and green practices in order to maximize the positive effects minimizing the environmental impact of the technology themselves. Standard procedures and policies should be developed to ensure the right trade-off between the benefits and the environmental impact of ICTs.

## Footnotes

1
https://brainteaser.health/open-evaluation-challenges/

## Footnotes

2
https://www.greendigitalcoalition.eu/
